# A significant quantitative trait locus on chromosome Z and its impact on egg production traits in seven maternal lines of meat-type chicken

**DOI:** 10.1186/s40104-022-00744-w

**Published:** 2022-08-09

**Authors:** Jiqiang Ding, Fan Ying, Qinghe Li, Gaomeng Zhang, Jin Zhang, Ranran Liu, Maiqing Zheng, Jie Wen, Guiping Zhao

**Affiliations:** 1 Institute of Animal Sciences, Chinese Academy of Agricultural Sciences, Beijing, China; 2 MiLe Xinguang Agricultural and Animal Industrials Corporation, MiLe, China

**Keywords:** Candidate gene, Egg production, Genome-wide association study, Meat-type chicken, Selective sweep analysis

## Abstract

**Background:**

Egg production is economically important in the meat-type chicken industry. To better understand the molecular genetic mechanism of egg production in meat-type chicken, genetic parameter estimation, genome-wide association analyses combined with meta-analyses, Bayesian analyses, and selective sweep analyses were performed to screen single nucleotide polymorphisms (SNPs) and other genetic loci that were significantly associated with egg number traits in 11,279 chickens from seven material lines.

**Results:**

Yellow-feathered meat-type chickens laid 115 eggs at 43 weeks of age and white-feathered chickens laid 143 eggs at 60 weeks of age, with heritability ranging from 0.034–0.258. Based on meta-analyses and selective sweep analyses, one region (10.81–13.05 Mb) on chromosome Z was associated with egg number in all lines. Further analyses using the W2 line was also associated with the same region, and 29 SNPs were identified that significantly affected estimation of breeding value of egg numbers. The 29 SNPs were identified as having a significant effect on the egg number EBV in 3194 birds in line W2. There are 36 genes in the region, with glial cell derived neurotrophic factor, DAB adaptor protein 2, protein kinase AMP-activated catalytic subunit alpha 1, NAD kinase 2, mitochondrial, WD repeat domain 70, leukemia inhibitory factor receptor alpha, complement C6, and complement C7 identified as being potentially affecting to egg number. In addition, three SNPs (rs318154184, rs13769886, and rs313325646) associated with egg number were located on or near the prolactin receptor gene.

**Conclusion:**

Our study used genomic information from different chicken lines and populations to identify a genomic region (spanning 2.24 Mb) associated with egg number. Nine genes and 29 SNPs were identified as the most likely candidate genes and variations for egg production. These results contribute to the identification of candidate genes and variants for egg traits in poultry.

**Supplementary Information:**

The online version contains supplementary material available at 10.1186/s40104-022-00744-w.

## Introduction

Throughout the twentieth century, specialized commercial chicken populations were established for egg laying and meat production [1]. Currently, layers display high levels of egg production and have been developed by intensive artificial selection in the egg-laying industry to yield more than 320 eggs during a 52-week laying period [2, 3]. Meat-type chickens have received substantial attention for their meat production, and to avoid selection for both growth traits and egg production traits in the same bird [1]. For meat-type chickens, the characteristics of body weight, growth rate, and feed conversion rate have been widely studied [4].

Although significant genetic changes may have occurred during selective breeding practices, the relationships between genotypes and phenotypes are not well characterized [2]. Many studies have shown that the heritability of egg number production. For example, such heritability was 0.13–0.45 and 0.10–0.40 for egg number production in two commercial lines of White Leghorn hens [5]. In the past three decades, many studies have explored egg production at the genetic level. Recent studies of reproductive hormones have identified 31 candidate genes significantly associated with egg production, and there are 64 new candidate genes and 108 SNPs associated with oviposition performance based on genome sequencing analyses [6]. Over 440 quantitative trait loci (QTL) on the *Gallus gallus* chromosome (GGA) have been reported to be associated with egg number in chickens [7]. With the deeply sequencing and the mining of increasing SNPs, genome-wide association studies (GWAS) and selective sweep analyses have become powerful tools for detecting QTLs in many breeds of livestock and poultry [8].

The growth and feed conversion traits of meat-type chickens have been widely reported, however, egg-laying traits have been less studied. For layers, multiple SNPs and QTLs have been found [6], but due to the genetic differences between layers and meat-type chickens, the application of those findings in meat-type chickens has been limited. Mapping of QTLs and SNPs with potential impact on egg production in meat-type chickens to provide more reference information for subsequent genome selection. Our experimental design involved GWAS and selective sweep analyses to detect genetic variations associated with egg number based on a 55 K SNP Chicken chip in a population of 11,279 meat-type chickens.

## Materials and methods

### Animals and phenotypes

A total of 11,279 meat-type chickens were obtained from seven lines of two breeding companies in China. The W1, W2, W3, and W4 were four commercial white-feathered pure lines in Yunnan Province, China. Body weight exceeded 2.4 kg at 40 days of age in lines W1, W2, and W4. Body weight exceeded 1.6 kg at 40 days of age in line W3. The feed conversion ratio was under 1.69 between 28 and 40 days of age, and the age at first egg was 175–180 d. The Y1, Y2, and Y3 were three commercial yellow-feathered pure lines in Jiangsu Province, China. The feed conversion ratio was ~ 3.13, which was considered medium growth rate meat-type chickens.

Line W2 was selected for the egg production more than eight generations. We used the individuals of three generations (3159 chickens: 2532 females; 627 males), including the 6th, 7th and 8th generations. All other lines were used for individual data of one generation. All chickens had pedigree, genomic information and were housed in identical individual cages. There were 11,279 individuals in all lines, of which 9279 were recorded for egg numbers. Blood samples were collected from the brachial veins of each chicken using the standard procedure of the breeding program, which was approved by the Animal Welfare and Ethics Committee of the Institute of Animal Sciences, Chinese Academy of Agricultural Sciences (IAS-CAAS, Beijing, China).

### Staged egg numbers statistics

We made a detailed division of the laying period using 627 hens from the 7th generation of line W2. We recorded the age at first egg (AFE), and egg production for each hen from first egg to 354 days of age. We divided the egg-laying period into four stages according to the laying curve in Additional file 1: Fig. S1 and counted the egg numbers (EN) at each stage. Four stages, including the stage of rapidly increasing egg production (from the onset of laying eggs to 195 days of age; EN1); the stage of peak egg production (from 196 to 227 days of age; EN2); the stage at which the laying rate was between 60% and 75% (from 228 to 307 days of age; EN3); and the stage of decreasing egg production (from 308 to 354 days of age; EN4). Moreover, we counted the total egg number from first egg to 354 d (TEN).

### Genotyping and quality control

Genomic DNA was extracted from blood samples using the phenol-chloroform method. All the birds were genotyped using a customized 55 K SNP chicken array obtained from Beijing Compass Biotechnology Co., Ltd. (Beijing, China) [9]. We updated the SNP positions using the newest release from the University of California-Santa Cruz (GRCg6a/galGal6 genome version). The following quality control criteria were applied to the target panel: individual call rate ≥ 90%, SNP call rate ≥ 90%, and minor allele frequency ≥ 0.05. Ultimately, 37,847–45,346 SNPs and 11,279 birds remained for further analyses.

### Population structure testing

Twenty individuals randomly selected from each line for structure using ADMIXTURE software (version1.3.0) [10]. A principal component analysis (PCA) plot was constructed using the “scatterplot3d” package in R (version 4.0.2).

### Genome-wide association study

GWAS was performed using 9778 individuals with egg number phenotypes from the all lines. The GWAS for egg number traits was performed using the univariate linear mixed model implemented in GEMMA software, version 0.98.1 [11]. The statistical model was as follows:

1 $$y= W\alpha + x\beta +u+\varepsilon; u\sim MV{N}_n\left(0,\lambda {\tau}^{-1}K\right), \varepsilon \sim MV{N}_n\left(0,{\tau}^{-1}{\mathrm{I}}_n\right)$$

where *y* is the vector of phenotypic values; *W* is the vector of covariates, including a column of 1 s; *α* is the vector of the corresponding coefficients, including the intercept; *x* is the vector of marker genotypes; *β* is the effect size of the marker; *u* is the vector of random polygenic effects; *ε* is the vector of errors; *τ*^−1^ is the variance of the residual errors; *λ* is the ratio between the two variance components; *K* is the centered relatedness matrix estimated from SNPs, and Ι_*n*_ is the identity matrix. *MVN*_*n*_ is the *n*-dimensional multivariate normal distribution. The Wald test was used to select SNPs associated with egg production traits.

We used the parameters of plink software --indep-pairwise 25 5 0.2 to infer effective independent tests. Considering the over-conservation of the 5% Bonferroni correction method, we adjusted the threshold of genome-wide significant *P*-values based on the number of independent SNPs [12]. Manhattan and Q-Q plots were constructed for each trait using the “qqman” package in R (version 4.0.2). Boxplots were produced by the “ggplot2” package in R (version 4.0.2).

### Meta-analyses

Meta-analyses of the seven lines were performed using standard method by Metal software (version 2011-03-25). Meta-analyses can combine either (a) test statistics and standard errors or (b) *P*-values across multiple GWAS for a single trait (taking sample size and direction of effect into account) [13]. The detailed calculation formula is introduced in reference [13]. Meta-analysis results in little or no loss of efficiency compared to analysis of a combined dataset including data from all individual studies.

### Association study on GGA Z

Due to the particularity of GGA Z, we used “-method threshold” function of SNPTEST v2.5.6 software [14], a software for sex chromosome association study, for GGA Z association study. For chickens, female genotypes are encoded as 0/1 in SNPTAST. In addition to association test statistics, SNPTEST can output expected genotype and allele counts for diploid and haploid samples. Computation of allele frequencies and info statistics also take into account ploidy.

### Selective sweep analysis

Selective sweep analysis was performed on all hens from the line W1 and 782 hens from the 8th generation of line W2, which had genomic information obtained by 55 K SNP chips. Line W2 was selected for the egg number more than eight generations, Line W1 was not selected for egg number. We used the “--weir-fst-pop” function of the Vcftools v0.1.14 software to calculate the population differentiation index (Fst), which were a window length of 40 kb and a step size of 2 kb [15].

### Bayesian analysis

Two Bayesian approaches, Bayes B and Bayes R, were used to obtain the estimated marker effect explained by each SNP. Bayes B and Bayes R analyses were performed using the BGLR and hibayes package in R, respectively. The number of iterations after the burn-in phase and that of the burn-in period were 20,000 and 10,000, respectively. The purpose of the Bayesian analysis was to estimate whether the QTL we mapped had significantly larger effects. The Bayesian analysis’ result was the effect size of the SNPs, while the result of GWAS was the *P* value and effect size of SNPs associated with phenotype. Their results could be verified by each other.

### Genome prediction models

The genome prediction models, genomic best line prediction (GBLUP) and single-step GBLUP (ssGBLUP), were utilized in this study and were as follows:

2 $$y= Xb+ Z\alpha +e$$

Where *y* is the vector of phenotypic value, *X* and *Z* are the association matrices of fixed and additive genetic effects, *b* is the vector of the fixed effect, *α* is the vector of the random additive genetic effect, and *e* is the vector of the random residual error. For GBLUP, when an *α* obeyed the following normal distribution *α* ~N(0, *G* ${\upsigma}_{\mathrm{u}}^2$), G was the consanguinity matrix (G matrix). In ssGBLUP, the H matrix replaces the G matrix.

H matrix construction:

3 $${H}^{-1}={A}^{-1}+\left[\begin{array}{cc}0& 0\\ {}0& {G}^{-1}-{A}_{22}^{-1}\end{array}\right]$$

Where *H* is an inter-individual relational matrix using genome-wide markers and pedigrees, *A* is the pedigree-based relatedness matrix, *G* is the genome-based relationship matrix, and *A*_22_ is the individual genealogical relation matrix of sequencing individuals. The relative weights of *G* and *A*_22_ in the H matrix were set as *G*_*ω*_ = 0.95 × *G* + 0.05 × *A*_22_ . The level of the *G* matrix was corrected to the *A*_22_ (matrix)

4 $${G}^{\ast }=a+b\times G$$

*G*^∗ ^represents the adjusted *G* matrix, and *a* and *b* are as follows:

5 $$\mathrm{Avg}\left(\operatorname{diag}(G)\right)\times b+a=\mathrm{Avg}\left(\operatorname{diag}\left({A}_{22}\right)\right)$$

6 $$\mathrm{Avg}\left(\mathrm{offdiag}(G)\right)\times b+a=\mathrm{Avg}\left(\mathrm{offdiag}\left({A}_{22}\right)\right)$$

The formula of the *H* matrix after merging is:

7 $${H}^{-1}={A}^{-1}+\left[\begin{array}{cc}0& 0\\ {}0& {\left[0.95\times \left(a+b\times G\right)+0.05\times {A}_{22}\right]}^{-1}-{A}_{22}^{-1}\end{array}\right]$$

The animal models of the two traits are as follows:

8 $$\left[\begin{array}{c}{y}_1\\ {}{y}_2\end{array}\right]=\left[\begin{array}{cc}{X}_1& 0\\ {}0& {X}_2\end{array}\right]\left[\begin{array}{c}{b}_1\\ {}{b}_2\end{array}\right]+\left[\begin{array}{cc}{Z}_1& 0\\ {}0& {Z}_2\end{array}\right]\left[\begin{array}{c}{\alpha}_1\\ {}{\alpha}_2\end{array}\right]+\left[\begin{array}{c}{e}_1\\ {}{e}_2\end{array}\right]$$

Where *y*, *X*, *b*, *Z*, *α*, and *e* are the same formula (2).

The genetic parameters of line W2 for egg production traits were calculated using the ssGBLUP model, and the heritability of the seven lines was estimated by the GBLUP model. GBLUP and ssGBLUP were calculated using ASReml v4.1 software [16].

## Results

### Basic statistics and genetic parameters

A total of 9778 hens from seven lines were subjected to genome-wide association analyses. The egg number of each line was evaluated at a different time. The heritability of egg number varied between the seven lines. The results showed that the egg number traits exhibited low heritability (0.034–0.258) (Table 1).

**Table 1:** Basic statistics for egg production traits in the seven lines

Lines^a^	EN^b^	n^c^	Age^d^	Heritability^e^	SNP^f^ N	Indep^g^ SNP	Significance^h^	Suggestive^i^
Y1	110.7	1653	43 weeks	0.156(0.038)	40,343	12,732	3.90E-06	7.90E-05
Y2	115.2	1582	43 weeks	0.173(0.034)	39,592	18,437	2.70E-06	5.40E-05
Y3	77.8	1437	43 weeks	0.257(0.046)	37,847	13,703	3.60E-06	7.30E-05
W1	53.1	224	34 weeks	0.034(0.143)	43,906	8132	6.10E-06	1.20E-04
W2	111.4	2532	50 weeks	0.153(0.064)	38,657	9756	5.10E-06	1.00E-04
W3	143.4	1135	60 weeks	0.258(0.043)	43,411	12,428	4.00E-06	8.00E-05
W4	122.5	1215	51 weeks	0.163(0.054)	52,343	12,480	4.00E-06	8.00E-05

^a^Y1, Y2, and Y3 represent three lines of yellow-feathered meat-type chickens; W1, W2, W3, and W4 represent four lines of white-feathered meat-type chickens

^b^EN represents egg number

^c^Number of individuals with the phenotype

^d^The week of age of egg number recorded

^e^heritability (standard error, SE)

^f^number of SNPs used in this study

^g^number of SNPs remaining after effective independent tests

^h^the *P*-value threshold for a genome-wide significance

^i^the *P*-value threshold for a genome-wide suggestive significance

The basic statistics for the distribution of egg production traits from the 627 hens used in the GWAS and genetic parameters are shown in Table 2. EN1 and EN4 had the largest phenotypic variation, as some chickens were late to laying and laid eggs at inconsistent intervals. Additionally, some birds died or were too old to lay eggs in EN4 because of their low rate of follicle development [17]. A total of 7771 independent tests of all chromosomal SNPs were performed. Therefore, the threshold *P*-values for genome-wide suggestive and genome-wide significance were calculated as 1.29E-04 (1.00/7771) and 6.43E-06 (0.05/7771), respectively.

**Table 2:** Descriptive statistics for egg production traits in 627 hens of line W2

Traits	Mean	SD	CV, %	Min	Max
AFE	174.75	7.94	4.55	159	200
EN1	16.15	6.93	42.89	1	36
EN2	25.42	3.92	15.41	2	51
EN3	54.97	10.23	18.61	5	80
EN4	28.21	8.71	30.86	1	47
TEN	122.94	23.51	19.12	42	180

AFE represents age at first egg; EN1, EN2, EN3, EN4, and TEN represent total egg numbers in each of the five stages (from onset of laying eggs to 195 d, from 196 to 227 d, from 228 to 307 d, from 308 to 354 d, and from the onset of laying eggs to 354 d, respectively); SD represents standard deviation; CV represents coefficient of variation

The heritability and correlation of egg production traits was estimated using a relationship matrix composed of pedigrees and genotypes. AFE and EN1 had medium heritability (0.24 and 0.27), while the heritability of ENs in other egg-laying stages was low, ranging from 0.09 to 0.17. AFE had a high negative correlation with EN1, a moderately negative correlation with TEN, and a low correlation with ENs in other egg-laying stages. It was shown that AFE affected the laying performance by affecting the EN1. EN1 had a moderately positive correlation with TEN, and low positive correlations with ENs in other egg-laying stages. EN2, EN3, EN4, and TEN exhibited high correlations with each other (> 0.8) (Table 3).

**Table 3:** Estimates of genetic parameters for egg production traits in 627 hens of line W2

Traits	AFE	EN1	EN2	EN3	EN4	TEN
AFE	**0.24(0.054)**	−0.96(0.020)	0.011(0.19)	0.025(0.22)	−0.0187(0.20)	−0.37(0.18)
EN1	−0.88(0.0058	**0.27(0.055)**	0.19(0.18)	0.14(0.21)	0.16(0.19)	0.50(0.16)
EN2	−0.086(0.026)	0.20(0.025)	**0.17(0.051)**	0.87(0.12)	0.70(0.15)	0.81(0.11)
EN3	0.023(0.026)	0.080(0.026)	0.51(0.019)	**0.093(0.038)**	0.77(0.13)	0.85(0.072)
EN4	0.013(0.026)	0.058(0.026)	0.35(0.023)	0.66(0.015)	**0.14(0.044)**	0.87(0.072)
TEN	−0.26(0.024)	0.39(0.022)	0.60(0.016)	0.88(0.0059)	0.82(0.0086)	**0.12(0.043)**

Heritability on the diagonal, genetic correlations above the diagonal and phenotypic correlations down the diagonal, SE in parentheses. AFE represents age at first egg; EN1, EN2, EN3, EN4, and TEN represent total egg numbers in each of the five stages (from onset of laying eggs to 195 d, from 196 to 227 d, from 228 to 307 d, from 308 to 354 d and from onset of laying eggs to 354 d, respectively)

### Population structure testing

**Fig. 1:**
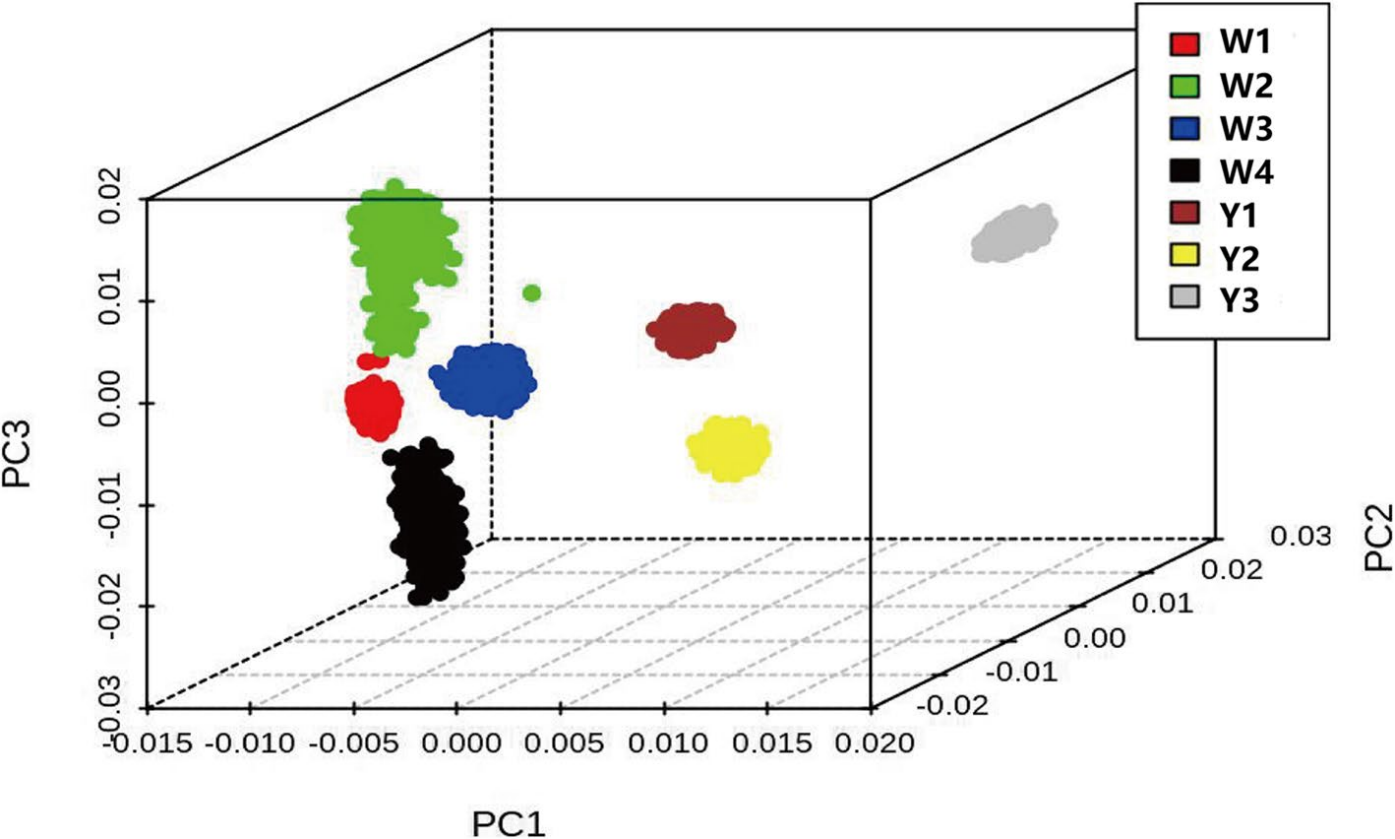
PCA plots for the seven lines. The first three PCs were used to calculate the genetic relationships between the seven lines. Y1, Y2, and Y3 represent three lines of yellow-feathered meat-type chickens, W1, W2, W3 and W4 represent four lines of white-feathered meat-type chickens. The four white-feathered meat-type chicken lines were closely related, and three yellow-feathered meat-type chicken lines 101, 401 and 501 were closely related

PCA using the first three principal components showed obvious population stratification between the seven lines and that four lines (W1, W2, W3, and W4) of white-feathered meat-type chickens were mixed with each other (Fig. 1).

Figure S2 displays a bar plot based of the cross-validation error rate. When K = 2, the fast-growing white-feathered and the yellow-feathered meat-type chickens appeared as two differentiated clusters. When K = 3, the four white-feathered meat-type chicken lines were divided into three groups. When K = 4, the three yellow-feathered populations were separated (Additional file 3: Fig. S3).

### GWAS for single-line egg number traits

GWAS was performed on each line, and 35 SNPs were associated with egg number. Using the Ensembl database to annotate SNPs, we found 31 genes around significant SNPs (Additional file 6: Table S1). For the GWAS of the seven lines, the genomic inflation factors (λ, 0.993–1.069) were calculated to evaluate the accuracy and reliability of the GWAS results (Additional file 4: Fig. S4).

### Meta-analysis based on GWAS results of single-line egg number traits

**Fig. 2:**
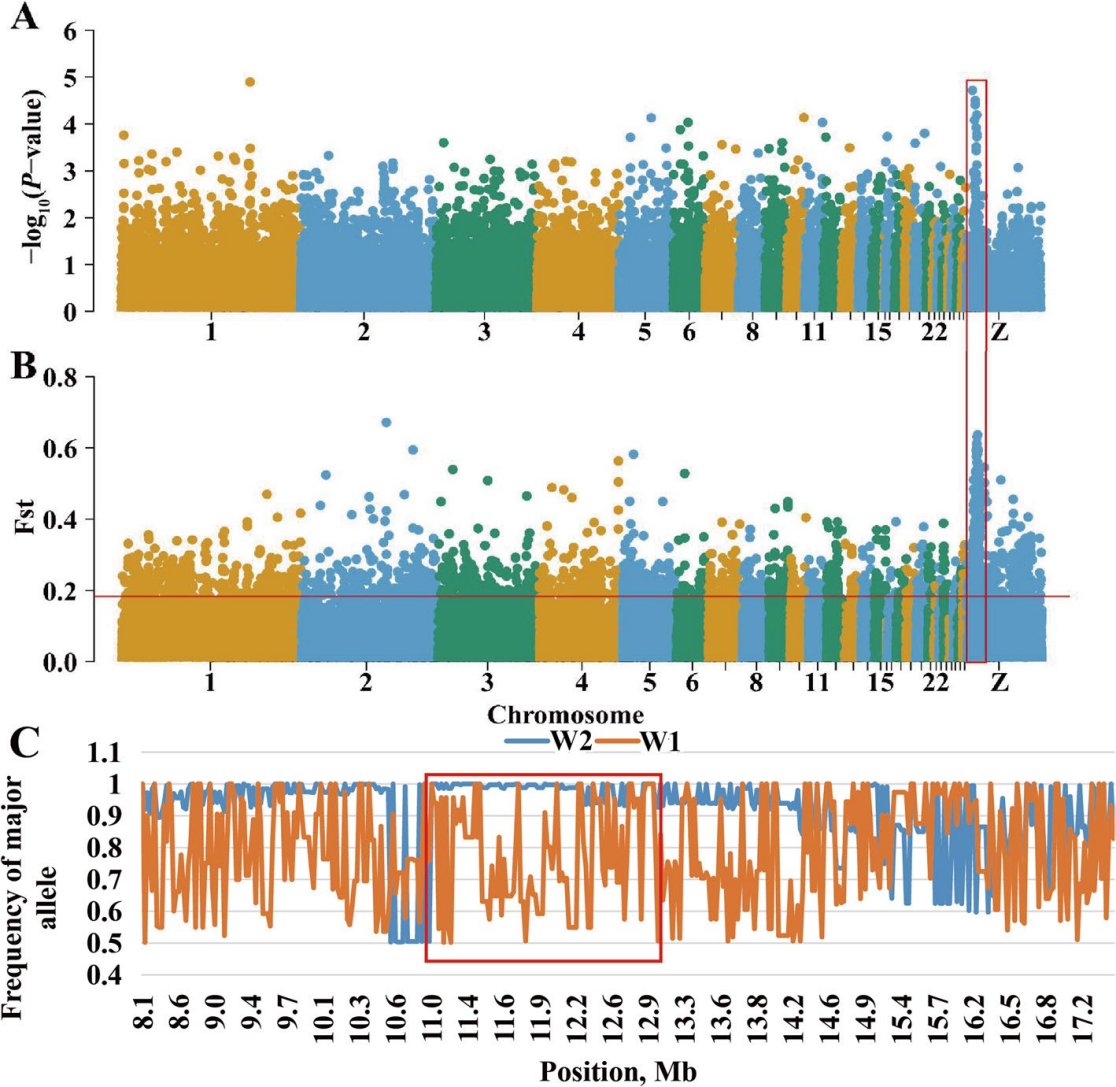
Selective sweep analyses and meta-analyses were located in a region of the GGA Z. **A** Manhattan plots of the meta-analyses for egg numbers of the seven lines; each dot represents one SNP. **B** The visualization of Fst in lines W1 and W2; each dot represents a calculation step size of 2 kb. **C** The major allele frequency analyses of SNPs located in the candidate region for lines W1 and W2

Through the above analysis, the SNP effect of egg numbers in a single line could be obtained, but it was unknown whether the same region exists for multiple lines with large genetic background differences. Therefore, using the above GWAS results, a meta-analysis was performed on all individuals of the seven lines, and an obvious bulge region was found on the Z chromosome (Fig. 2A).

### Significant region was differentiated between lines with different breeding directions

The Fst and major allele frequency analyses were performed on the line W1 and W2 chickens (Fig. 2B and C). It was a significant signal of selection that a QTL region overlapped with the result of meta-analysis located between 10.81 Mb and 13.05 Mb on the GGA Z. The region contains 36 genes. Eight genes were identified as candidate genes after functional annotation. The genes included glial cell derived neurotrophic factor (*GDNF*), DAB adaptor protein 2 (*DAB2*), protein kinase AMP-activated catalytic subunit alpha 1 (*PRKAA1)*, NAD kinase 2, mitochondrial (*NADK2*), WD repeat domain 70 (*WDR70*), leukemia inhibitory factor receptor alpha (*LIFR*), complement C6 (*C6*), and complement C7 (*C7*).

### Phased egg number traits to verify significant QTL region

1. GWAS for staged egg number traits

**Fig. 3:**
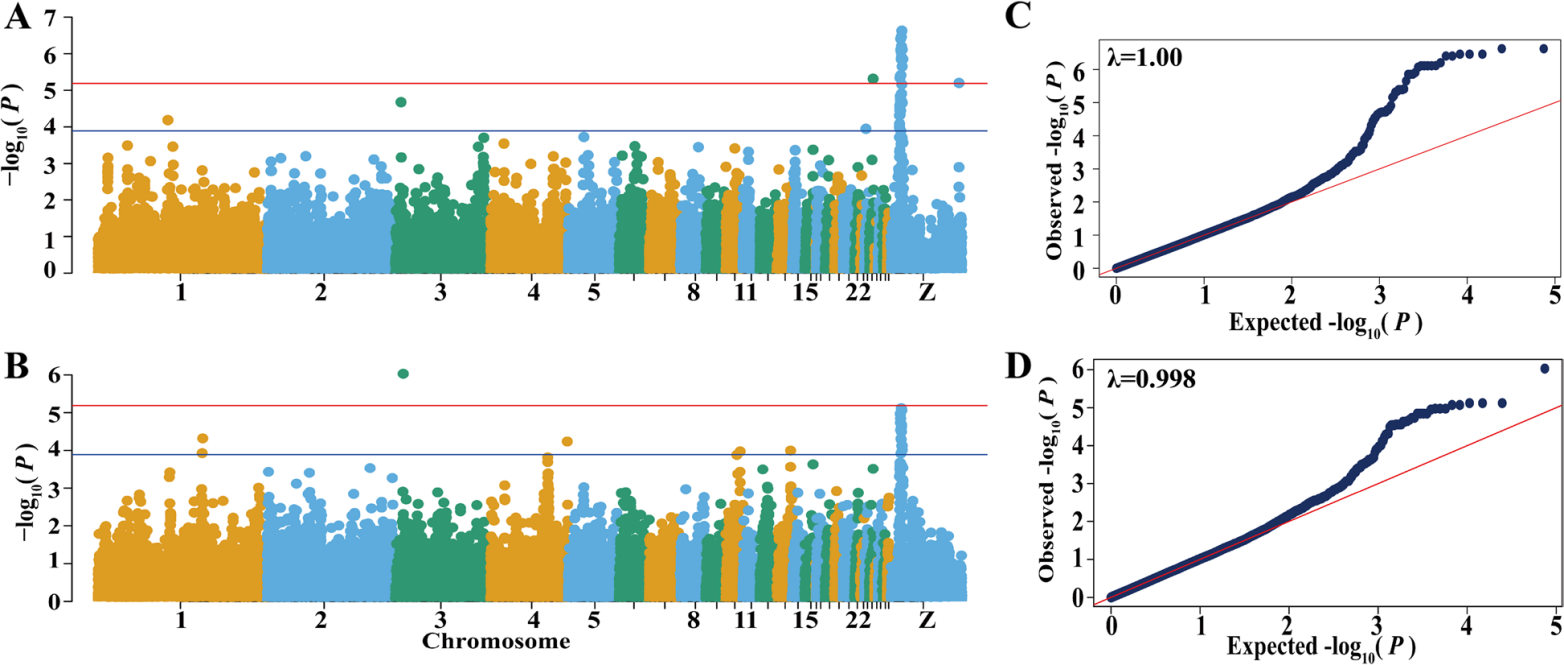
Manhattan and quantile–quantile plots of the GWAS for EN2 and TEN traits. Each dot represents a SNP in the dataset. The horizontal red and blue lines indicate the genome-wide significance (*P*-value = 6.43e-6) and suggestive thresholds (*P*-value = 1.29e−4), respectively. **A, C** Manhattan and quantile–quantile (Q-Q) plots of EN2 (egg number from 196 to 227 d); λ was the genomic inflation factor. **B, D** Manhattan and quantile–quantile (Q-Q) plots of TEN (egg number from onset of laying eggs to 354 d)

Combined with the results of GWAS and selective sweep analysis, we further analyzed the region between 10.81 Mb and 13.05 Mb on the GGA Z in line W2. For the GWAS of 627 hens in line W2, Manhattan and quantile–quantile (Q-Q) plots for egg production traits were generated, as shown in Fig. 3 and Additional file 5: Fig. S5. Detailed information on the SNPs significantly associated with EN2, EN3, EN4, and TEN is shown in Table 4. For the EN2, 26 genome-wide significant SNPs were identified on GGA Z, and 38 genome-wide suggestive SNPs were detected on GGA Z, GGA1, GGA3, GGA4, GGA10, and GGA13. The genomic inflation factor (*λ*) was 1.00 for EN2, which suggested that the population structure was well controlled.

**Table 4:** Annotation of SNPs with significance associated with egg number traits in 627 of line W2 hens

Traits^a^	rs	GGA^b^	Position^c^	Allele	AF	β^d^	*P*-value	Gene	Gap, kb^e^
EN2	rs315536207	24	4,203,575	G/A	0.41	−0.84	4.84E-06	*THY1*	Instron
EN2	rs316053320	Z	10,263,677	A/G	0.11	−1.00	4.60E-06	*ADAMTS12*	Instron
EN2, EN3, TEN	rs318154184	Z	10,661,667	T/A	0.46	1.97	4.08E-06	*PRLR*	Instron
EN2, EN3, TEN	rs13769886	Z	10,672,563	G/C	0.46	1.97	4.08E-06	*PRLR*	Instron
EN2, EN3, TEN	rs313325646	Z	10,706,318	A/G	0.46	1.97	4.08E-06	*PRLR*	U 26.6 KB
EN2, TEN	rs317829338	Z	11,039,011	C/A	0.11	−1.07	3.92E-07	*RANBP3L*	Instron
EN2, TEN	rs317316870	Z	11,049,964	C/G	0.11	−1.07	3.92E-07	*NADK2*	U 25.3 KB
EN2, TEN	rs16130829	Z	11,138,239	G/A	0.11	−1.04	7.72E-07	*SLC1A3*	D 34.0 KB
EN2, TEN	rs313925951	Z	11,181,202	A/G	0.11	−1.04	7.72E-07	*SLC1A3*	Instron
EN2, TEN	rs315242894	Z	11,250,945	G/A	0.11	−1.04	7.72E-07	*SLC1A3*	U 16.7 KB
EN2, TEN	rs315141712	Z	11,295,727	G/A	0.11	−1.04	7.72E-07	*SLC1A3*	D 58.6 KB
EN2, TEN	rs313907922	Z	11,336,593	A/G	0.1	−0.97	3.87E-06	*SLC1A3*	D 98.5 KB
EN2, TEN	rs314230546	Z	11,360,621	T/A	0.12	−1.03	8.51E-07	*CPLANE1*	D 106.3 KB
EN2, TEN	rs316797236	Z	11,583,068	T/A	0.09	−1.06	1.39E-06	*WDR70*	Instron
EN2, TEN	rs318193135	Z	11,775,041	A/G	0.09	−1.12	3.46E-07	*GDNF*	U 30.6 KB
EN2, TEN	rs14786300	Z	11,823,333	A/G	0.09	−1.12	3.46E-07	*GDNF*	U 77.9 KB
EN2, TEN	rs14786270	Z	11,831,103	G/A	0.09	−1.12	3.46E-07	*GDNF*	U 85.5 KB
EN2, TEN	rs14786427	Z	11,995,193	A/G	0.09	−1.13	6.28E-07	*EGFLM*	D 2.7 KB
EN2, TEN	rs317925374	Z	12,003,955	T/A	0.1	−1.03	1.23E-06	*LIFR*	D 3.33 KB
EN2, TEN	rs16131724	Z	12,481,249	A/G	0.12	−1.07	2.36E-07	*DAB2*	U 117.9 KB
EN2, TEN	rs317751736	Z	12,614,891	A/G	0.12	−1.07	2.36E-07	*OTGER4*	D 219.8 KB
EN2	rs14756086	Z	12,901,024	G/A	0.16	−0.84	2.22E-06	*PRKAA1*	Instron
EN2, TEN	rs317744265	Z	12,965,710	A/G	0.11	−1.03	7.63E-07	*C6*	D 0.7 KB
EN2	rs14756790	Z	13,656,667	A/G	0.13	−0.93	1.40E-06	*HMGCS1*	Instron
EN2	rs316455929	Z	13,662,376	A/G	0.13	−0.93	1.40E-06	*HMGCS1*	Instron
EN2	rs313666070	Z	79,095,862	G/A	0.26	0.63	6.28E-06	*FEM1C*	D 22.0 KB
EN3	rs14485191	4	66,642,445	A/G	0.22	−3.06	3.29E-06	*CORIN*	Instron
EN3	rs318004733	4	66,681,294	G/A	0.22	−3	4.29E-06	*CORIN*	Instron
TEN, EN4	rs14319456	3	6,449,298	G/A	0.2	−0.92	2.11E-05	*NRXN1*	D 706.6 KB

^a^EN2, EN3, EN4, and TEN represent egg numbers at four stages (from 196 to 227 d, from 228 to 307 d, from 308 to 354 d, and from onset of laying eggs to 354 d, respectively)

^b^*Gallus gallus* chromosome

^c^Gallus_gallus-6.0 source

^d^Allele substitution effect was the additive effect estimated by GEMMA

^e^U and D indicate that the SNP is upstream and downstream of a gene, respectively

**Fig. 4:**
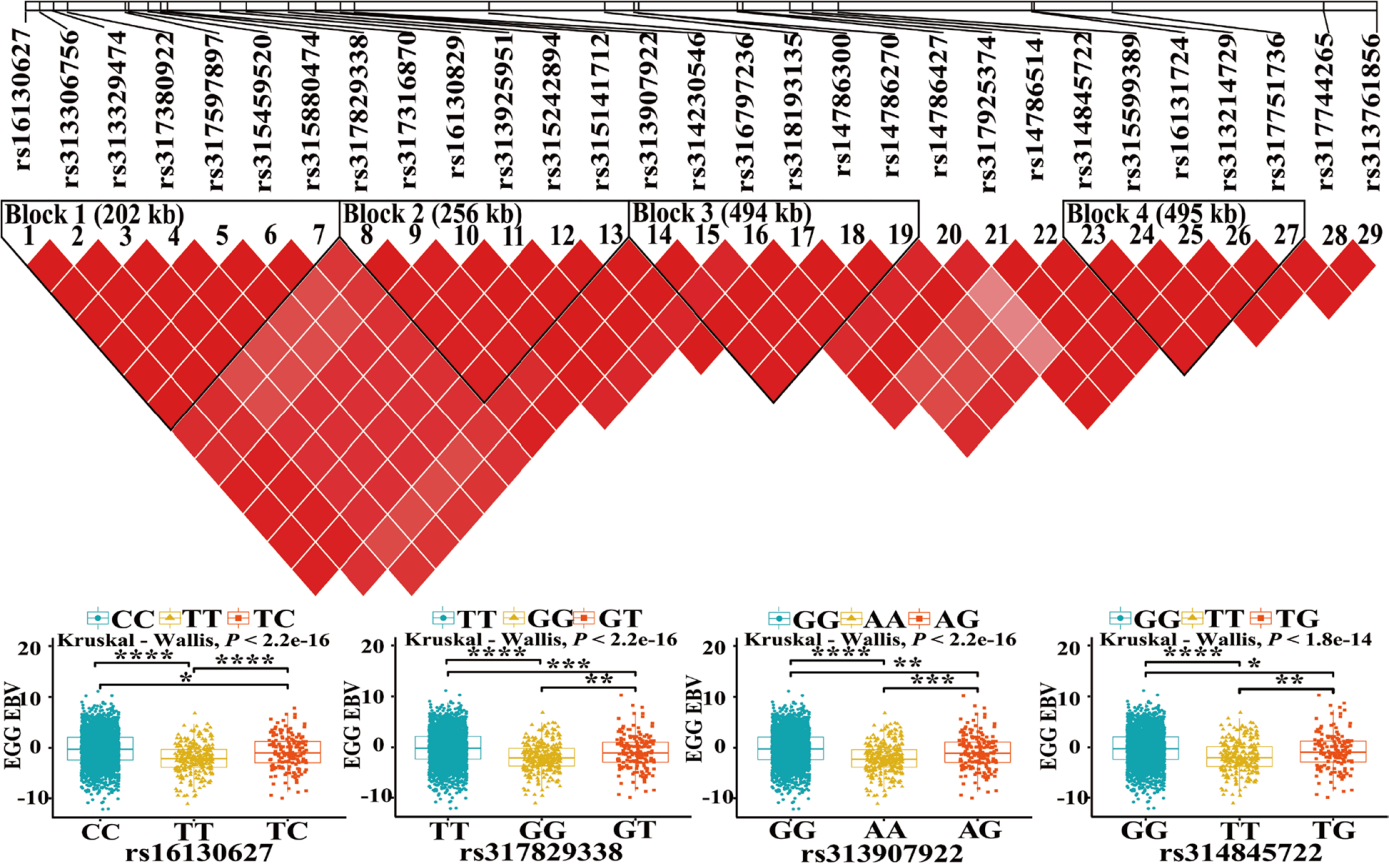
LD plots of the 29 significant SNPs from 3159 chickens in line W2. Box plots were structured using four SNPs selected from four blocks. The SNPs effect was tested using Kruskal-Wallis univariate ANOVA analyses for egg number EBV

For the TEN, one significant SNP and 38 suggestive SNPs were detected on GGA Z, GGA1, GGA3, and GGA23. The SNPs on the GGA Z were also associated with EN2. Three SNPs (rs318154184, rs13769886, rs313325646) associated with EN2, EN3 and TEN, were located on or near the prolactin receptor (*PRLR*) gene on the GGA Z. There were 33 coincidently associated SNPs for EN2 and TEN between 10.81 Mb and 13.05 Mb on GGA Z, among which 29 SNPs were associated with significantly different egg number EBVs of the 3159 individuals in line W2. The 29 SNPs were located in four LD blocks (Fig. 4), and the visualization for analysis of variance of one SNP selected each Block.

(2) Association tests on GGA Z

**Fig. 5:**
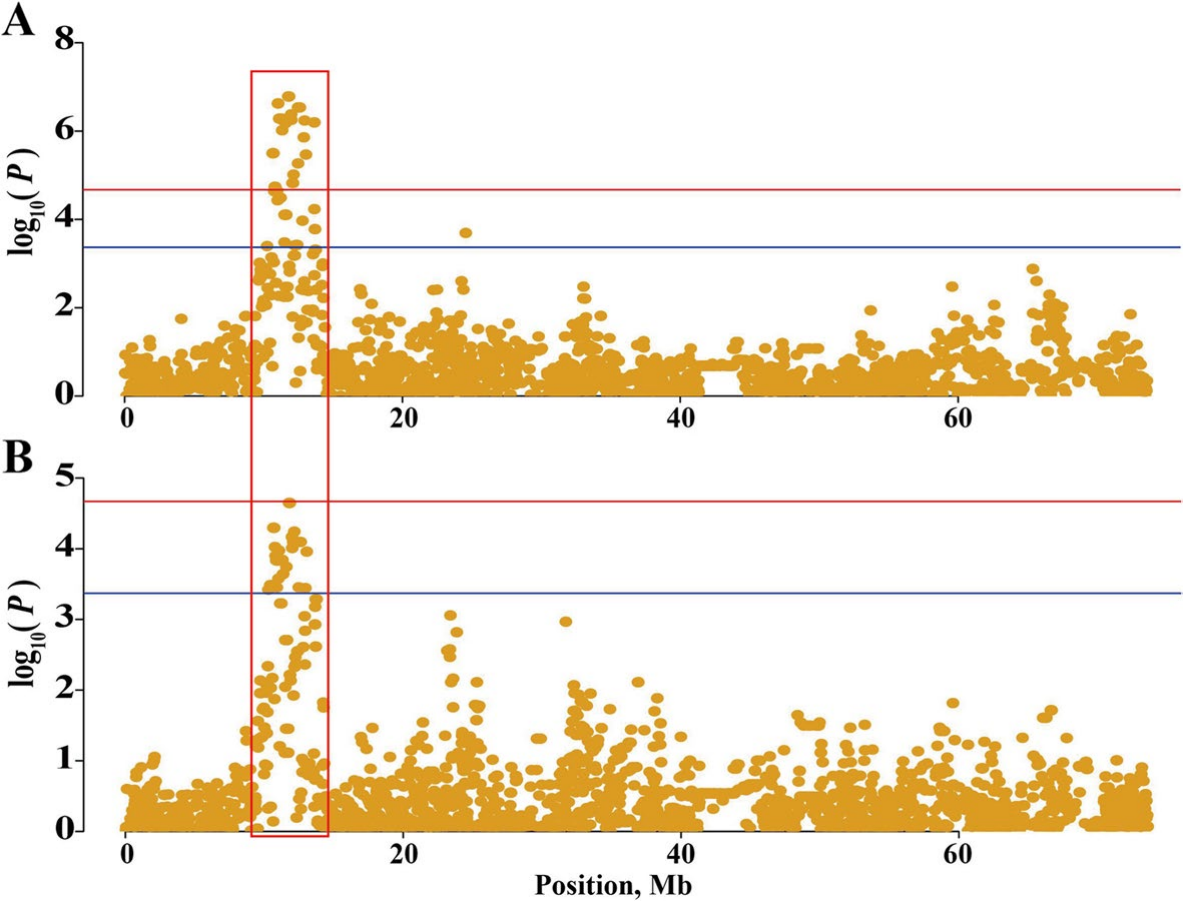
Manhattan plots of GGA Z association study for EN2 and TEN traits. Each dot represents a SNP in the dataset. The horizontal red and blue lines indicate the genome-wide significance (*P*-value = 2.14e-5) and suggestive thresholds (*P*-value = 4.26e− 4), respectively. **A** Manhattan plot of EN2 (egg number from 196 to 227 d); **B** Manhattan plot of TEN (egg number from onset of laying eggs to 354 d)

Considering the particularity of GGA Z, we used SNPTEST software to analyze GGA Z of EN2 and TEN traits of 627 hens in line W2 specially. The results (Fig. 5) were consistent with those in (1), which could indicate the reliability of the region between 10.81 Mb and 13.05 Mb on GGA Z.

(3) The effect of each SNP

We used Bayes B and Bayes R to estimate the effect value of each SNP using the EN2 and TEN phenotypes of 627 hens from line W2. There were obvious bulges in the significant QTL region, which was similar to GEMMA in that the calculated effect size also had obvious bulges in the region (Additional file 6: Fig. S6). In the effect estimated for this region, GEMMA, Bayes B and Bayes R estimated EN2 and TEN accounted for 0.89%, 1.01%, 0.47%, 0.37%, 0.52%, and 0.52% of total effects, respectively. This result indicated that there were indeed strong effects in the region that could affect phenotypic variation.

### Significance analysis between phenotypes and SNPs of the QTL region

**Fig. 6:**
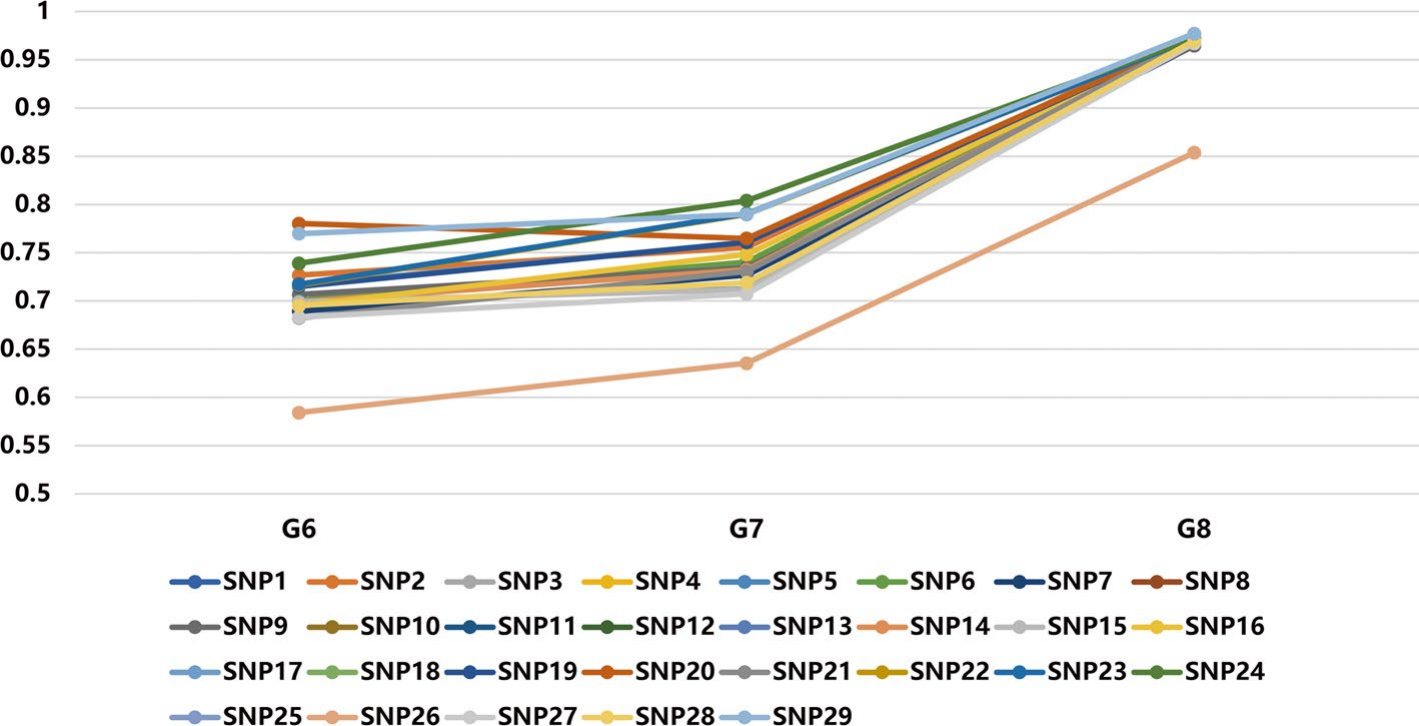
Frequency variations of the 29 SNPs associated with higher egg number EBVs. G6, G7, and G8 represent the three generations of the line W2 chickens, and each line represents the differences in the frequency change curve of a SNP’ genotype with higher egg number EBV

To further verify the correlation between egg numbers and 29 SNPs, we analyzed the SNPs’ change of dominant genotypes with breeding. We found that the frequency of dominant genotypes gradually increased with the selection of W2 for egg production, which indicated that the SNPs were also selected with the selection of phenotype, which could well explain these SNPs and egg production is significantly correlated (Fig. 6). Therefore, the 29 SNPs were identified as important candidate SNPs (Table 5).

**Table 5:** Annotation of the 29 SNPs that were significantly different in EBV of 3159 individuals

GGA^a^	rs	Position^b^	Alleles	*P*-value	Candidate gene	Distance, kb^c^
Z	rs16130627	10,815,822	A/G	1.65E-05	*SPEF2*	Intron
Z	rs313306756	10,839,079	G/A	1.65E-05	*SPEF2*	Exon1
Z	rs313329474	10,861,996	A/G	1.90E-05	*ENSGALG00000051958*	Intron
Z	rs317380922	10,885,055	C/G	1.90E-05	*IL7R*	U 1.9
Z	rs317597897	10,980,433	G/A	1.90E-05	*SKP2*	Exon2
Z	rs315459520	10,985,955	G/A	1.90E-05	*SKP2*	Intron
Z	rs315880474	11,018,303	G/A	3.60E-05	*NADK2*	Intron
Z	rs317829338	11,039,011	C/A	3.92E-07	*RANBP3L*	Intron
Z	rs317316870	11,049,964	C/G	3.92E-07	*RANBP3L*	Exon1
Z	rs16130829	11,138,239	G/A	7.72E-07	*SLC1A3*	D 32.3
Z	rs313925951	11,181,202	A/G	7.72E-07	*SLC1A3*	Intron
Z	rs315242894	11,250,945	G/A	7.72E-07	*SLC1A3*	D 16.03
Z	rs315141712	11,295,727	G/A	7.72E-07	*SLC1A3*	D 60.82
Z	rs313907922	11,336,593	A/G	3.87E-06	*ENSGALG00000003605*	U 21.55
Z	rs314230546	11,360,621	T/A	8.51E-07	*ENSGALG00000003605*	Intron
Z	rs316797236	11,583,068	T/A	1.39E-06	*WDR70*	Intron
Z	rs318193135	11,775,041	A/G	3.46E-07	*GDNF*	D 32.13
Z	rs14786300	11,823,333	A/G	3.46E-07	*GDNF*	D 80.42
Z	rs14786270	11,831,103	G/A	3.46E-07	*GDNF*	D 88.19
Z	rs14786427	11,995,193	A/G	6.28E-07	*EGFLAM*	D 2.04
Z	rs317925374	12,003,955	T/A	1.23E-06	*LIFR*	D 3.48
Z	rs14786514	12,081,685	G/A	1.98E-05	*LIFR*	U 24.65
Z	rs314845722	12,119,113	A/C	1.98E-05	*ENSGALG00000048563*	U 32.25
Z	rs315599389	12,161,567	T/A	1.23E-05	*ENSGALG00000048563*	Intron
Z	rs16131724	12,481,249	A/G	2.36E-07	*DAB2*	D 110.09
Z	rs313214729	12,485,174	A/G	6.92E-06	*DAB2*	D 114.02
Z	rs317751736	12,614,891	A/G	2.36E-07	*ENSGALG00000048297*	D 5.73
Z	rs317744265	12,965,710	A/G	7.63E-07	*C6*	D 0.38
Z	rs313761856	13,053,866	A/C	2.20E-05	*PLCXD3*	Intron

^a^G*allus gallus* chromosome

^b^Gallus_gallus-6.0 source

^c^U and D indicate that the SNP is upstream and downstream of a gene, respectively

## Discussion

In the twentieth century, layers and meat-type chickens were developed in different directions to avoid conflicts between egg-producing and meat-producing traits [18]. Such activities were extremely successful in improving productivity; laying hens produced more than 320 eggs during their 52-week laying period, while broilers gained 50–60 times their weight from hatching to sale [19]. Those activities also led to substantial genomic differences between layers and meat-type chickens. To explore the genetic mechanisms of laying traits in meat-type chickens, 11,279 chickens were used in this experiment.

The genetic contributions of egg number have low or medium levels of heritability, typically ranging from 0.16 to 0.64 [6]. However, among the seven lines evaluated in this study, the heritability of egg number was different (0.034–0.258), but it was not the color of their feathers that caused the difference. The range of heritability observed in this study was consistent with those reported in White Leghorn hens (0.05–0.27) [20]. The main reasons for other differences in heritability included environmental effects, population size, animal breeds, differences in assessment methods, and the reliability of the individual information records.

Further analysis the EN of partial periods and the total period in Line W2, Liu et al. predicted heritability range between 0.05–0.24 for White Leghorn hens [12]. Our results showed that AFE and EN1 had a high level (0.24–0.27) of heritability, with the heritability of the other stages being 0.093–0.17, which was slightly lower than those previously reported (0.17–0.20) [21]. AFE had a strong negative genetic correlation with EN1 (− 0.96), and a moderate negative correlation with TEN (− 0.37), which was consistent with the findings of Yuan et al. [21] and Liu et al. [12].

Meta-analyses have been widely used in multi-population GWAS studies. Falker-Gieske et al. used 1306 White Leghorn chickens and a meta-analysis of GWASs to identify the variations and genes related to feather pecking, and found 15 significant SNPs that may be related to feather pecking [22].

We used GWAS, meta-analyses, selective sweep analyses, and Bayesian analyses to scan the SNPs and QTLs associated with laying traits in meat-type chickens. A 10.81–13.05 Mb region on GGA Z was analyzed using these methods and 36 genes were annotated in this region. However, no studies have detailed the genes related to egg number traits in chickens. Notably, several regions associated with egg number traits have been identified on the GGA Z (https://www.animalgenome.org/cgi-bin/QTLdb/GG/index). The regions identified in our study were 1.9 Mb apart from those reported by Zhao et al., which were carried out in more than a dozen breeds, including White Leghorn and Rhode Island Red chickens [23]. Although the results were different between the present study and that of Zhao et al., they suggest that GGA Z is an important genetic region involved in egg production.

Referring to prior studies, we found that the regions between 10.81 Mb and 13.05 Mb on the GGA Z identified in this study were inconsistent with those reported in layers, which may be due to the differences in the genomes between layers and meat-type chickens. In the regions, genes that may be involved in reproductive traits were identified, including the *GDNF* gene*,* which has been reported to affect the in vitro maturation of human oocytes, increasing the prevalence of MII stage oocytes from 34% to 49% [24]. *GDNF* has also been reported to affect the development of oocytes in a variety of animals, such as humans, pigs and mice [25–27].

DAB2 is an endocytic adaptor protein in several NPXY motif-containing cell surface receptors, including the lipoprotein receptor and integrin. Budna et al. also suggested that the *DAB2* gene may be involve in the regulation of MII phase oocyte formation and other processes critical to porcine fertility [28]. The *PRKAA1* gene encodes the catalytic α-subunit of 5′ AMP-activated protein kinase has an opposite effect--maintenance of the meiotic block in porcine and bovine oocytes [29, 30] AMPK can control steroidogenesis in ovarian cells (granulosa, theca cells and corpus luteum cells) and germ cell maturation [31]. The *NADK2* gene in this region, the NAD kinase (NADK) is the sole NADP biosynthetic enzyme [32]. NADPH, which is the reduced form of NADP, regenerates cellular oxidative defense systems via glutathione reductase to counteract oxidative damage [33, 34]. Therefore, NADK2 may affect egg production by affecting reactive oxygen species levels [34]. The *WDR70* gene was identified as a candidate gene for milk production and reproductive traits in Chinese and Northern European Holstein dairy cows [35]. The gene is homologous in chickens and cattle and may have the same function [36].

The *LIFR* gene can form polymeric complexes with other receptors, such as glycoprotein 130 (GP130), to stimulate the JAK/STAT, MAPK and PI3K signaling pathways [37]. JAK/STAT and PI3K signaling pathways influence the hypothalamic-pituitary-ovarian (HPO) axis to regulate the breeding cycle of laying hens. Two genes, *C6* and *C7,* were identified in this region. The complement system plays an important role in the process of inflammation and infection, and also provides a link between innate and adaptive immunities. Immune capacity can affect the reproductive performance, health, and welfare of hens [38].

In addition, three SNPs (rs318154184, rs13769886, rs313325646) associated with EN2, EN3, and TEN were located on or near the prolactin receptor (*PRLR*) gene on GGA Z. The *PRLR* gene is associated with laying traits and consists of 15 exons and 14 introns in chicken [39, 40]. Liu et al. hypothesized that *PRLR* might be the primary gene responsible for sexual maturation and is considered a candidate genetic marker for reproductive traits [41]. Studies showed that the prolactin gene is expressed in the hypothalamus, pituitary, oviduct, and ovary of chickens. Some studies also showed that polymorphisms occur at different locations of the *PRLR* gene, including SNP in exons 2, 5 [40], and 10 [39], which were mainly associated with egg number traits.

## Conclusion

In conclusion, our results indicated that the heritability of egg numbers was different in different chicken populations, and egg number was a low-heritability trait. Based on GWAS analyses of 627 individuals in one generation of line W2, meta-analyses of seven lines, and selective sweep analyses of lines W1 and W2, we found an interesting region on GGA Z. Where multiple SNPs and genes may be significantly related to egg production. Our findings contributed to a better understanding of the genetic basis of egg production.

## Supplementary information

**Additional file 1: Fig. S1.** Egg production curve of the complete laying period.

**Additional file 2: Fig. S2.** Line chart of cross validation error.

**Additional file 3: Fig. S3.** Admixture plot.

**Additional file 4: Fig. S4.** Manhattan and quantile–quantile (Q-Q) plots of the GWAS for egg number of 7 lines.

**Additional file 5: Fig. S5.** Manhattan and quantile–quantile (Q-Q) plots of the GWAS for AFE, EN1, EN3, and EN4 traits in line W2 chickens.

**Additional file 6: Fig. S6.** Manhattan plots of the estimated squared-marker effect from GWAS, Bayes B and Bayes R.

**Additional file 7: Table S1.** Annotation of significant SNPs associated with the seven chicken lines.

**Additional file 8.** The phenotypic data used in this study.

## Data Availability

The whole genome sequencing data reported in this paper were deposited in the Figshare data center under accession number 10.6084/m9.figshare.19178906 that can be publicly accessed at https://figshare.com/.

## Abbreviations

GGA: *Gallus gallus* chromosome
GWAS: Genome-wide association study
LD: Linkage disequilibrium
QTL: Quantitative trait locus
Animal QTLdb: Animal QTL Database
SNP: Single nucleotide polymorphism
AFE: The age at first egg
EN: Egg numbers
EN1: The egg number from the onset of laying eggs to 195 days of age
EN2: The egg number from 196 to 227 days of age
EN3: The egg number from 228 to 307 days of age
EN4: The egg number from 308 to 354 days of age
TEN: The total egg number from first egg to 354 d
PCA: Principal component analysis
Q-Q: Quantile–quantile
Fst: The population differentiation index
GBLUP: Genomic best line prediction
ssGBLUP: Single-step GBLUP
GDNF: Glial cell derived neurotrophic factor
DAB2: DAB adaptor protein 2
PRKAA1: Protein kinase AMP-activated catalytic subunit alpha 1
NADK2: NAD kinase 2, mitochondrial
WDR70: WD repeat domain 70
LIFR: Leukemia inhibitory factor receptor alpha
C6: Complement C6
C7: Complement C7
PRLR: Prolactin receptor
AMPK: Adenosine 5′-monophosphate (AMP)-activated protein kinase
