# Supplementary figures and images for: A significant quantitative trait locus on chromosome Z and its impact on egg production traits in seven maternal lines of meat-type chicken
Source: J Anim Sci Biotechnol. 2022 Aug 9;13:96. doi: 10.1186/s40104-022-00744-w (PMC9361671; doi:10.1186/s40104-022-00744-w)

Fig. S1 Egg production curve of the complete laying period

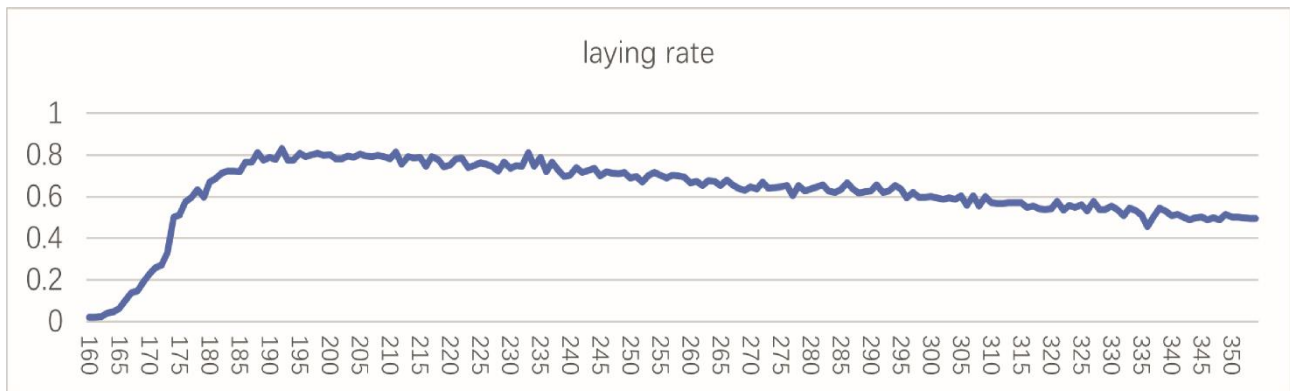

Supplement: Supplementary file 1 — Additional file 1: Fig. S1. Egg production curve of the complete laying period. [file 40104_2022_744_MOESM1_ESM.pdf]

Fig. S3. Admixture plot

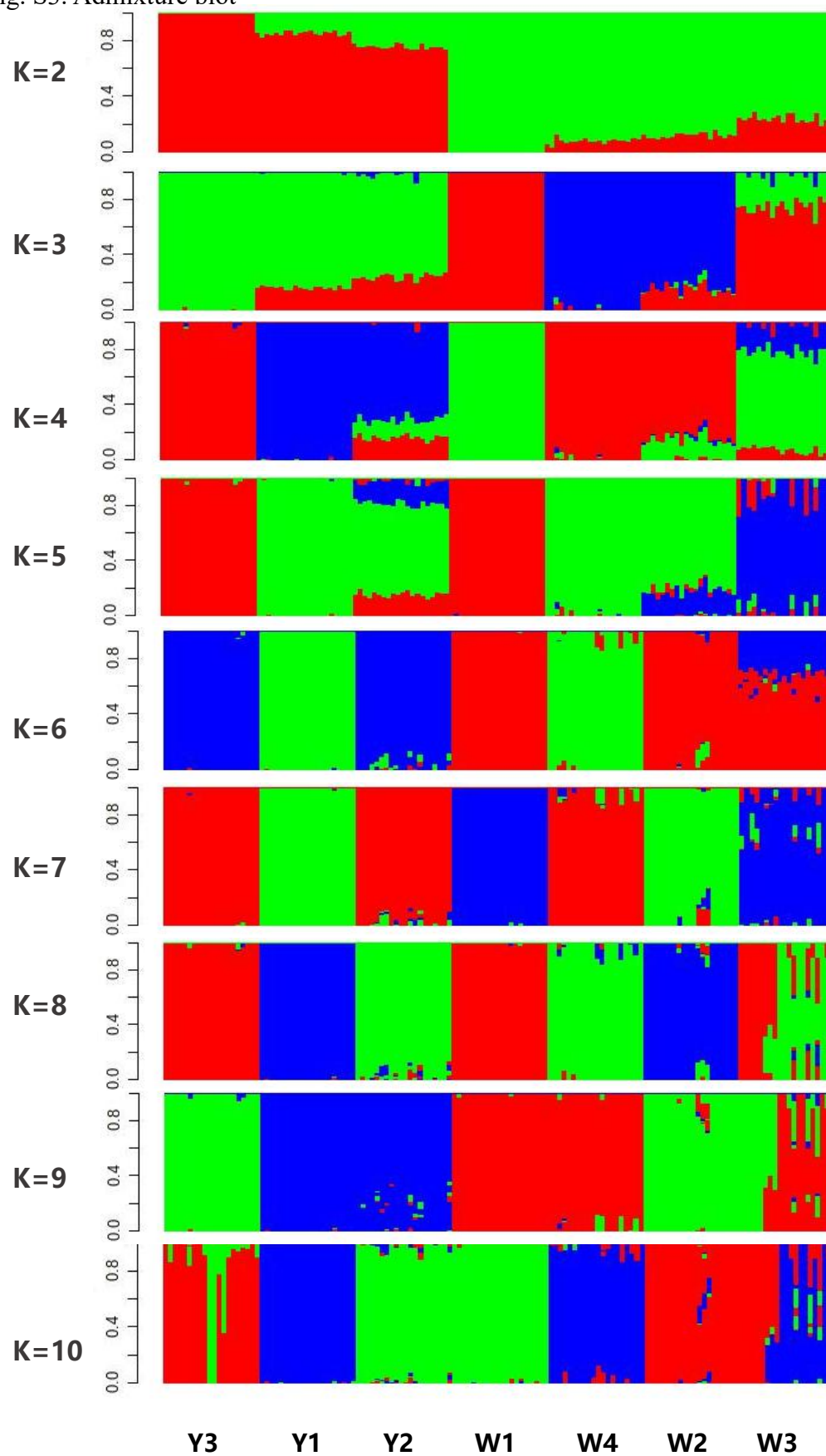

Supplement: Supplementary file 3 — Additional file 3: Fig. S3. Admixture plot. [file 40104_2022_744_MOESM3_ESM.pdf]
