# Supplementary material for: A significant quantitative trait locus on chromosome Z and its impact on egg production traits in seven maternal lines of meat-type chicken
Source: J Anim Sci Biotechnol. 2022 Aug 9;13:96. doi: 10.1186/s40104-022-00744-w (PMC9361671; doi:10.1186/s40104-022-00744-w)
Supplement: Supplementary file 2 — Additional file 2: Fig. S2. Line chart of cross validation error. [file 40104_2022_744_MOESM2_ESM.pdf]

Fig. S2 The line chart of cross validation error.

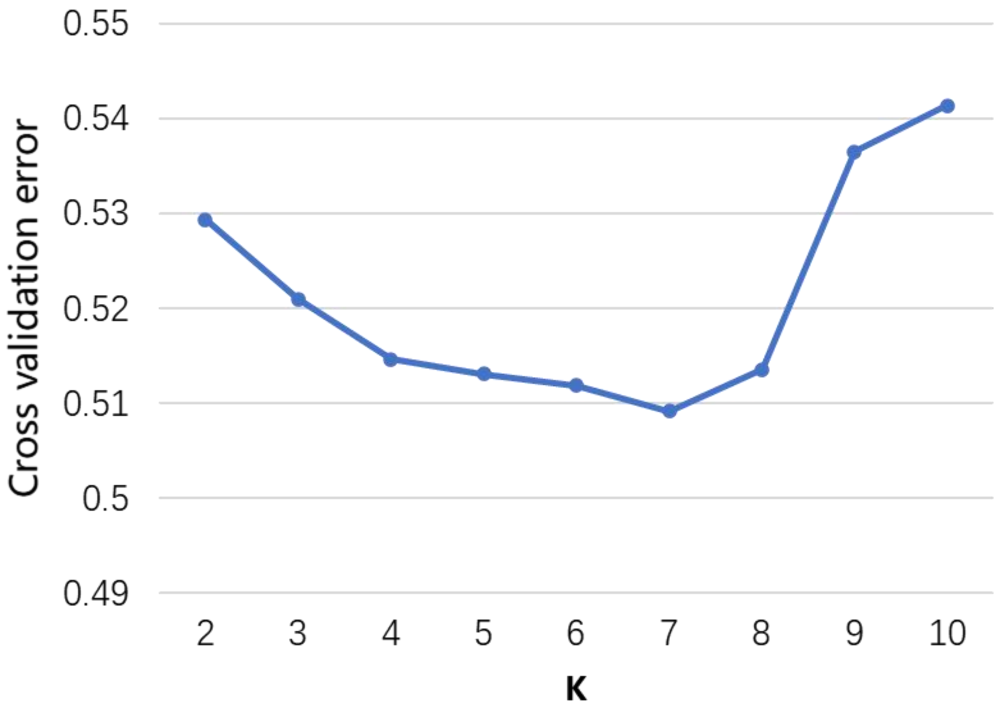

The coefficient of variation value for each K-value, the accessions were divided into ten subgroups (there was minimum K-value when K=7).
