# Supplementary material for: A significant quantitative trait locus on chromosome Z and its impact on egg production traits in seven maternal lines of meat-type chicken
Source: J Anim Sci Biotechnol. 2022 Aug 9;13:96. doi: 10.1186/s40104-022-00744-w (PMC9361671; doi:10.1186/s40104-022-00744-w)
Supplement: Supplementary file 4 — Additional file 4: Fig. S4. Manhattan and quantile–quantile (Q-Q) plots of the GWAS for egg number of 7 lines. [file 40104_2022_744_MOESM4_ESM.pdf]

Fig. S4 Manhattan and quantile–quantile (Q-Q) plots of the GWAS for egg number of 7 lines.

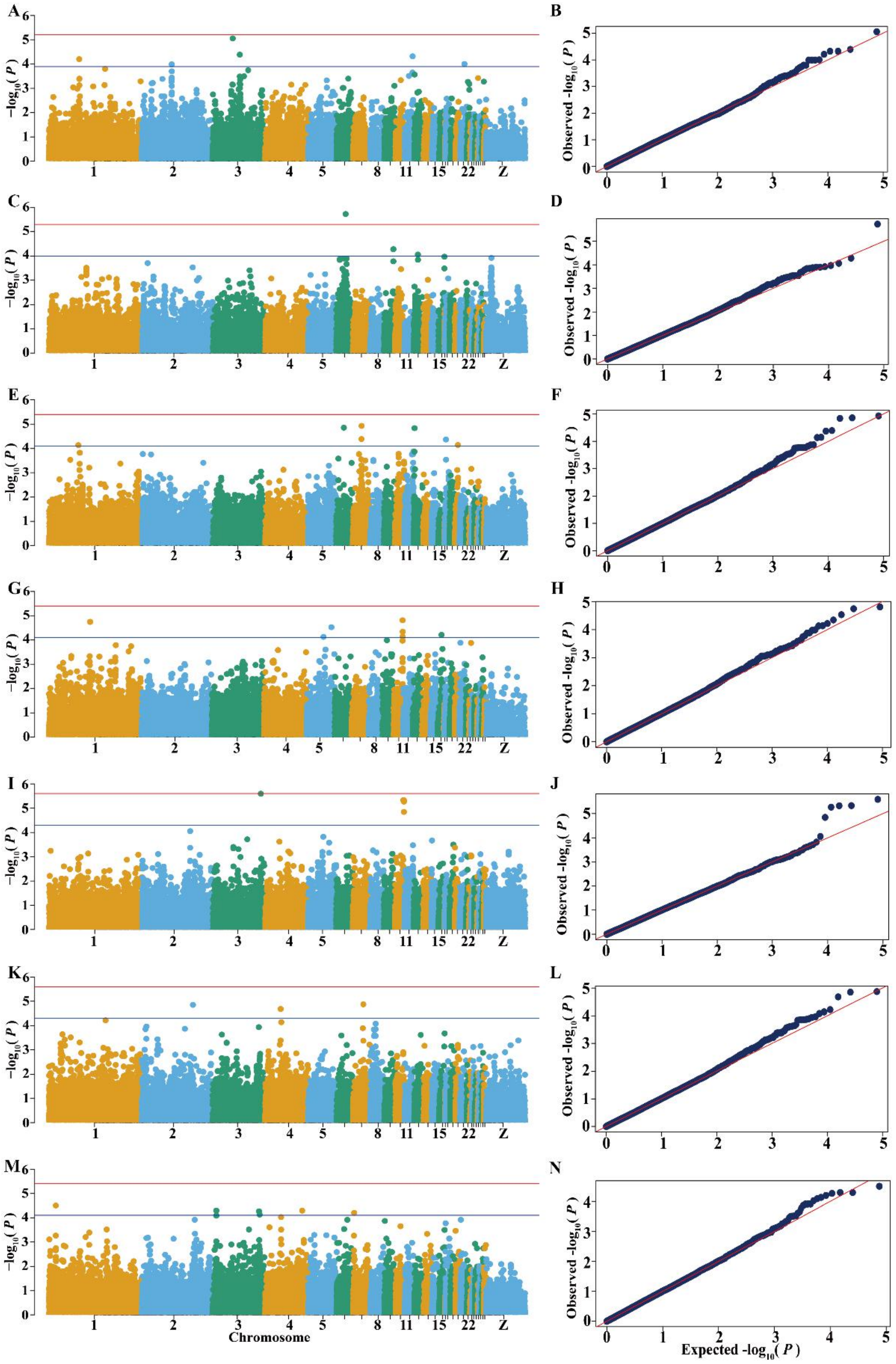

A, C, E, G, I, K and M represent the Manhattan plots of lines W1, W2, W3, W4, Y1, Y2 and Y3, respectively; In the Manhattan plots each dot represents a SNP; The red and blue horizontal lines represent genome-wide significance thresholds and potential

suggestive thresholds. B, D, F, H, J, L and N represent the Quantile–quantile plots of lines W1, W2, W3, W4, Y1, Y2 and Y3, respectively; The red line represents the concordance of observed and expected values. And the genomic inflation factor (λ) for each line is as follows: Y1, $\lambda = 0.993$; Y2, $\lambda = 0.997$; Y3, $\lambda = 0.997$; W1, $\lambda = 1.069$; W2, $\lambda = 0.988$; W3, $\lambda = 0.983$; W4, $\lambda = 1.026$. These results demonstrate the high accuracy and reliability of the GWAS results.
