# Supplementary material for: A significant quantitative trait locus on chromosome Z and its impact on egg production traits in seven maternal lines of meat-type chicken
Source: J Anim Sci Biotechnol. 2022 Aug 9;13:96. doi: 10.1186/s40104-022-00744-w (PMC9361671; doi:10.1186/s40104-022-00744-w)
Supplement: Supplementary file 5 — Additional file 5: Fig. S5. Manhattan and quantile–quantile (Q-Q) plots of the GWAS for AFE, EN1, EN3, and EN4 traits in line W2 chickens. [file 40104_2022_744_MOESM5_ESM.pdf]

Figure.S5 Manhattan and quantile–quantile (Q-Q) plots of the GWAS for AFE, EN1, EN3and EN4 traits.

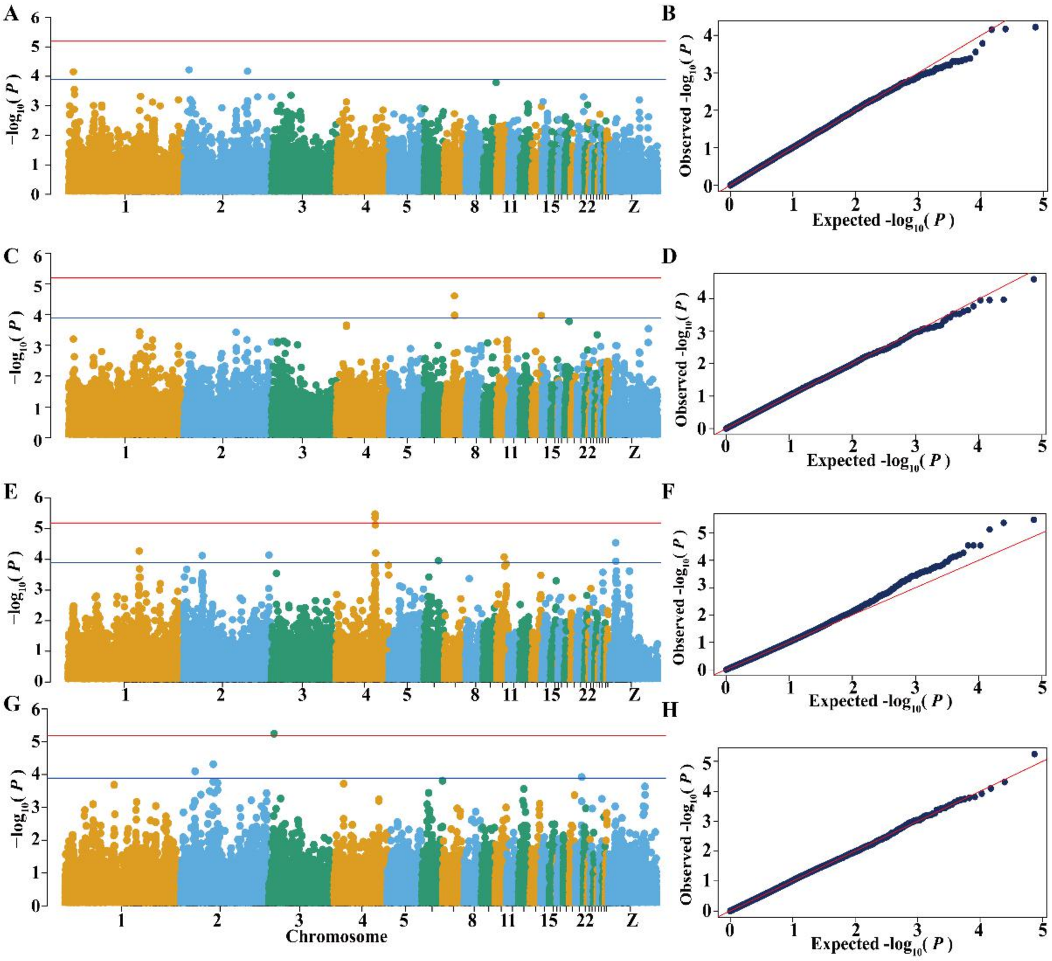

A, C, E and G represent the Manhattan plots of AFE, EN1, EN3 and EN4, respectively; Each dot is a SNP in the dataset; The horizontal red and blue lines indicate the genome-wide significant (*P* value =6.43e-6) and suggestive thresholds (*P* value = 1.29e−4), respectively. B, D, F and H represent the Quantile–quantile plots of AFE, EN1, EN3 and EN4, respectively; The red line represents the concordance of observed and expected values. And the genomic inflation factor (λ) for each line is as follows: AFE, λ = 1.04; EN1, λ = 1.04; EN3, λ = 0.998; EN4, λ = 0.993. These results demonstrate the high accuracy and reliability of the GWAS results.

The AFE represents the age at first egg; EN1, EN3, and EN4 represent egg numbers at four stages (egg number from onset of laying eggs to 195 days, from 228 to 307 days, and from 308 to 354 days, respectively)
