# Supplementary material for: A significant quantitative trait locus on chromosome Z and its impact on egg production traits in seven maternal lines of meat-type chicken
Source: J Anim Sci Biotechnol. 2022 Aug 9;13:96. doi: 10.1186/s40104-022-00744-w (PMC9361671; doi:10.1186/s40104-022-00744-w)
Supplement: Supplementary file 6 — Additional file 6: Fig. S6. Manhattan plots of the estimated squared-marker effect from GWAS, Bayes B and Bayes R. [file 40104_2022_744_MOESM6_ESM.pdf]

Fig. S6 Manhattan plots of the estimated squared-marker effect from GWAS, Bayes B and Bayes R.

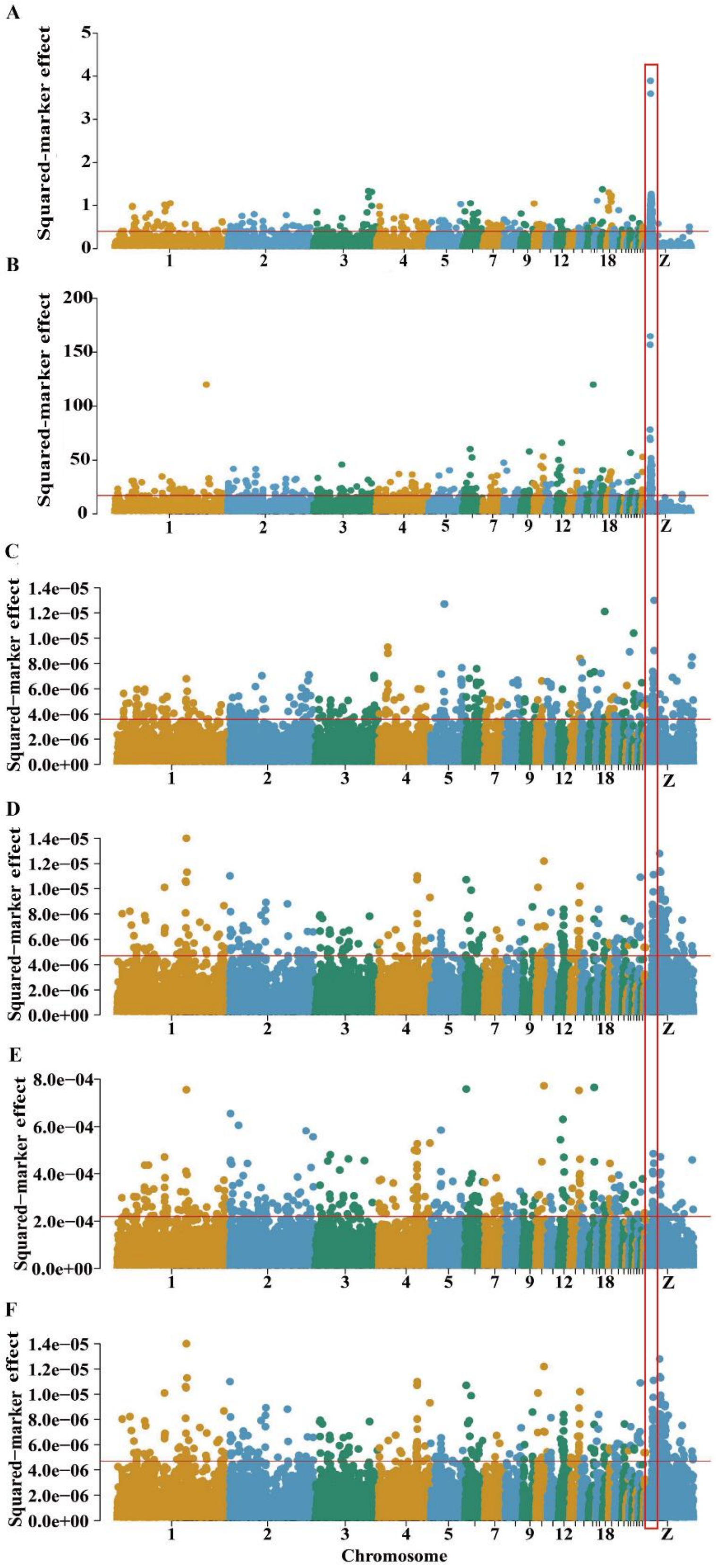

Legends: (A) (B) represent GWAS for EN2 and TEN, (C) (D) (E) (F) represent Bayes B analyses for EN2, Bayes R analyses for EN2, Bayes B analyses for TEN, and Bayes R analyses for TEN, respectively.
