# Supplementary material for: A significant quantitative trait locus on chromosome Z and its impact on egg production traits in seven maternal lines of meat-type chicken
Source: J Anim Sci Biotechnol. 2022 Aug 9;13:96. doi: 10.1186/s40104-022-00744-w (PMC9361671; doi:10.1186/s40104-022-00744-w)
Supplement: Supplementary file 7 — Additional file 7: Table S1. Annotation of significant SNPs associated with the seven chicken lines. [file 40104_2022_744_MOESM7_ESM.pdf]

Table S1. Annotation of significant SNPs associated with 7 lines.

Lines	rs	GGA[a]	Position[b]	β[c]	*P* value	Gene	Gap, kb[d]
Y1	rs14404973	3	101,343,319	2.61	2.56E-06	*ENSGALG00000016475*	intron
Y1	rs314600081	10	18,394,068	2.34	4.70E-06	*IGDCC4*	intron
Y1	rs312892904	10	19,796,467	-2.24	1.44E-05	*KIF23*	D 3.76
Y1	rs315407734	10	19,803,842	2.34	4.74E-06	*KIF23*	D 10.96
Y1	rs314663731	10	19,804,829	2.34	5.39E-06	*KIF23*	D 11.92
Y2	rs318061712	2	107,771,809	-3.18	1.41E-05	*SPIDR*	intron
Y2	rs317654235	4	32,260,081	-2.79	2.08E-05	*NR3C2*	intron
Y2	rs312789477	7	21,897,882	-2.86	1.34E-05	*LY75*	intron
Y3	rs315622159	1	14,368,997	-5.77	3.12E-05	*NAMPTP1*	intron
W1	rs317899003	1	63,942,199	6.87	6.23E-05	*ENSGALG00000053831*	intron
W1	rs316040498	2	62,981,592	-5.42	1.02E-04	*TMEM170B*	D 9.04
W1	rs317557970	2	62,983,613	-5.42	1.02E-04	*TMEM170B*	D 11.02
W1	rs16018073	2	62,989,847	-5.42	1.02E-04	*TMEM170B*	D 17.11
W1	rs314293804	3	42,424,680	-9.18	8.82E-06	*ENSGALG00000051089*	D 3.93
W1	rs315274567	3	57,379,107	7.54	4.07E-05	*MED23*	intron
W1	rs314851312	11	16,190,275	-8.14	4.76E-05	*CDH13*	intron
W1	rs14027438	11	16,197,749	-8.14	4.76E-05	*CDH13*	intron
W1	rs317528345	20	8,604,396	-9.83	9.90E-05	*ENSGALG00000046924*	intron
W2	rs14747990	6	20,750,628	-3.4	1.88E-06	*ENSGALG00000050084*	exon
W2	rs166 77209	9	20,459,639	-3.35	5.19E-05	*MECOM*	D 22.31
W2	rs316948110	12	9,191,205	2.75	8.60E-05	*SLMAP*	intron
W3	rs316386348	1	62,183,595	10.53	7.27E-05	*BPGM*	3 prime UTR
W3	rs315825336	6	17,092,252	-5.77	1.40E-05	*ENSGALG00000051819*	intron
W3	rs15855668	7	18,602,932	-8.16	4.09E-05	*ENSGALG00000044224*	U 0.85
W3	rs313721223	7	18,647,907	7.12	1.18E-05	*FASTKD1*	intron
W3	rs312667371	12	2,151,697	-5.97	1.46E-05	*ENSGALG00000048708*	U 0.52
W3	rs315486653	17	3,121,708	5.78	4.25E-05	*TNC*	U 22.98
W3	rs431881571	19	6,736,363	-5.65	7.15E-05	*ENSGALG0000004509*	U 1.68
W4	rs13897687	1	87,509,580	4.35	1.80E-05	*ENSGALG0000048148*	intron
W4	chr5:35782438	5	35,782,438	-16.99	7.39E-05	*NPAS3*	intron
W4	rs312322983	5	52,560,002	3.82	2.96E-05	*ENSGALG00000048544*	D 9.63
W4	rs313726052	10	19,251,303	-5.15	1.55E-05	*AAGAB/SMAD3*	intron
W4	rs733550169	10	19,456,463	-4.87	7.32E-05	*MAP2K5*	intron
W4	rs10721736	10	19,575,366	-4.92	4.52E-05	*CALML4*	3 prime UTR
W4	rs312625498	15	8,440,067	-3.84	6.08E-05	*CABIN1*	intron

a: Gallus gallus chromosome

b: Gallus_gallus-6.0 source

c: Allele substitution effect was the additive effect estimated by GEMMA

d: U and D indicate that the SNP is upstream and downstream of a gene, respectively
